# Herbal Medicine Treatment for Children with Autism Spectrum Disorder: A Systematic Review

**DOI:** 10.1155/2017/8614680

**Published:** 2017-05-16

**Authors:** Miran Bang, Sun Haeng Lee, Seung-Hun Cho, Sun-Ae Yu, Kibong Kim, Hsu Yuan Lu, Gyu Tae Chang, Sang Yeon Min

**Affiliations:** ^1^Department of Pediatrics of Korean Medicine, Graduate School of Dongguk University, Pildong-ro 1-Gil 30, Jung-gu, Seoul 04620, Republic of Korea; ^2^Department of Pediatrics of Korean Medicine, Kyung Hee University Korean Medical Hospital, Kyung Hee University Medical Center, Kyung Hee Dae-ro 23, Dongdaemun-gu, Seoul 02447, Republic of Korea; ^3^Department of Neuropsychiatry, College of Korean Medicine, Kyung Hee University, Kyung Hee Dae-ro 26, Dongdaemun-gu, Seoul 02447, Republic of Korea; ^4^Department of Pediatrics of Korean Medicine, College of Korean Medicine, Dongeui University, Yangjeong-ro 52-57, Busanjin-gu, Busan 47227, Republic of Korea; ^5^Department of Pediatrics, Korean Medicine Hospital, Pusan National University, Geumo-ro 20, Mulgeum-eup, Yangsan-si, Gyeongsangnam-do 50612, Republic of Korea; ^6^Chan-Nuri Hospital of Korean Medicine, Wonjeok-ro 469, Bupyeong-gu, Incheon 21365, Republic of Korea; ^7^Department of Pediatrics of Korean Medicine, Kyung Hee University Hospital at Gangdong, Dongnam-ro 892, Gangdong-gu, Seoul 05278, Republic of Korea; ^8^Department of Pediatrics of Korean Medicine, Korean Medicine Hospital, Dongguk University Medical Center, Dongguk-ro 27, Ilsandong-gu, Goyang-si, Gyeonggi-do 10326, Republic of Korea

## Abstract

**Objective:**

To summarize and evaluate the efficacy and safety of herbal medicines used for the treatment of autism spectrum disorder (ASD) in children.

**Methods:**

Thirteen electronic databases were searched from their inception to November 2016. Randomized controlled trials (RCTs) that assessed the efficacy of herbal medicines alone or in combination with other Traditional Chinese Medicine treatments for ASD in children were included. The Cochrane Risk of Bias Tool was used and other data analyses were performed using RevMan (Version 5.3).

**Results:**

Ten RCTs involving 567 patients with ASD were included for qualitative synthesis. In conjunction with conventional therapy, herbal medicines significantly improved the Childhood Autism Rating Scale (CARS) score, but the results of effects on total effective rate (TER) were different between the included studies. The use of herbal medicines with integrative therapy improved the CARS score and TER. In the studies that documented adverse events, no serious events were associated with herbal medicines.

**Conclusions:**

The efficacy of herbal medicines for the treatment of ASD appears to be encouraging but was inconclusive owing to low methodological quality, herbal medicine diversity, and small sample size of the examined studies.

## 1. Introduction

The core features of autism spectrum disorder (ASD) are persistent deficits in social communication and interaction and restricted, repetitive patterns of behavior, interests, or activities [1]. According to estimates from Center for Disease Control and Prevention (CDC) data, approximately 1 in 68 children has been identified with ASD. Studies in North America, Asia, and Europe have reported the average prevalence of individuals with autism as between 1% and 2% [2]. ASD is a lifelong condition of rising prevalence and community concern. The etiology of ASD is still controversial; various hypotheses concerning genetics, environmental factors, neurobiological factors, and neuropathology have been proffered [3].

There are many different types of treatment for ASD, such as medication management, education, rehabilitation training, sensory integration, and dietary approaches. Although there are no treatments for the core features of ASD, certain medications and behavioral therapies have been identified for the management of hyperactivity, depression, inattention, or seizures [4, 5]. Among the pharmacologic interventions, risperidone is the most commonly used treatment for serious behavioral symptoms in children with autism [6]. Despite its beneficial effects on behavioral problems, the results of risperidone treatment are inconclusive and have been associated with adverse events, such as increased appetite, rhinorrhea, somnolence, and excessive weight gain [7]. The parents of children with ASD are therefore concerned about potential adverse drug effects and are seeking treatments that are more secure. The volume of research into herbal medicines, a form of Complementary and Alternative Medicine (CAM), with fewer adverse effects, has increased for the treatment of children with ASD.

Herbal medicines and acupuncture are commonly used in the treatment of children with ASD [8]. There have been some systematic reviews of acupuncture [9–11], CAM [12, 13], and one review article of herbal medicines [8]. A systematic review on CAM for the treatment of ASD reported promising results for acupuncture, massage, music therapy, and sensory integration therapy [13]. All three systematic reviews of acupuncture concluded that further high quality trials were needed to evaluate the efficacy of acupuncture for autistic children [9–11] and one of these reviews suggested that acupuncture treatment showed behavioral and developmental improvements in children with ASD [11].

A review of herbal medicines reported that 32 kinds of Chinese herbal medicine have pharmacological effects, which mainly resulted in immune system improvement, memory enhancement, gastrointestinal tract improvement, and calming of the nerves [8]. However, that study did not provide evidence on the efficacy of the treatment of children with ASD. There is a lack of evidence on the efficacy of herbal medicines in the treatment of autistic children. The systematic review described here aimed to evaluate the clinical efficacy of herbal medicines as a treatment for ASD in children.

## 2. Methods

### 2.1. Data Source and Search Strategy

Databases and search terms were determined through discussion between all authors before the literature searches were executed; Sun Haeng Lee performed the electronic literature searches. The following electronic databases were searched for studies uploaded by November 2016 that investigated the treatment of ASD: MEDLINE, EMBASE, AMED, Cumulative Index to Nursing and Allied Health Literature (CINAHL), Cochrane Library, PsycARTICLES, three Korean databases (KoreaMed, Oriental Medicine Advanced Searching Integrated System (OASIS), and Korean Traditional Knowledge Portal (KTCKP)), two Chinese database (China National Knowledge Infrastructure (CNKI) and WanFang Data), and two Japanese databases (CiNii and Japanese Institutional Repositories Online (JAIRO)). The following search strategy was used in MEDLINE: (autis^*∗*^ OR pervasive developmental disorder^*∗*^ OR childhood disintegrative disorder OR Asperger^*∗*^ OR Autism Spectrum Disorder OR Child Development Disorders, Pervasive) AND (herb^*∗*^ OR decoction^*∗*^ OR remed^*∗*^ OR Chinese medic^*∗*^ OR Korean medi^*∗*^ OR kampo OR formul^*∗*^ OR herbal drug^*∗*^ OR Chinese drug^*∗*^ OR plant^*∗*^ OR Chinese prescrip^*∗*^ OR Chinese materica^*∗*^medica^*∗*^ OR traditional medic^*∗*^ OR Medicine, East Asian Traditional OR Herbal Medicine). To search the Korean, Chinese, and Japanese databases, slight modifications were applied to the above strategy. The details of search strategies used in English databases are presented in the Supplementary Material (Supplement  1, in Supplementary Material available online at https://doi.org/10.1155/2017/8614680). We contacted the original authors of the included studies via e-mail if additional information was needed. The protocol of this review was registered in PROSPERO (an international prospective register of systematic reviews) with the registration number CRD42016053391. The protocol of this review is available from https://www.crd.york.ac.uk/PROSPERO/display_record.asp?ID=CRD42016053391.

### 2.2. Inclusion Criteria

We only included randomized controlled trials (RCTs) that aimed to assess the efficacy of herbal medicines or herbal medicines in combination with other Traditional Chinese Medicine (TCM) treatments for ASD in children. The other TCM treatments included, but were not limited to, acupuncture, acupoint injection, Chuna therapy, and acupoint massage. RCTs were not limited to placebo-controlled, parallel-group, or cross-over studies. Other designs such as in vivo, in vitro, case reports, and retrospective studies were excluded. The herbal medicine forms (e.g., formula, decoction, and pills) were not restricted. Studies using herbal medicines in combination with conventional therapies, such as behavioral therapy, rehabilitation, education, and Western medicine, were included. All participants were aged less than 18 years and were diagnosed with ASD. The outcome measures of the trials were also restricted. The primary outcome measures included one or more of the following: Childhood Autism Rating Scale (CARS), Autism Behavior Checklist (ABC), and Aberrant Behavior Checklist-Community (ABC-C). The secondary outcome measures included total effective rate (TER) determined based on the improvement of clinical symptoms and the reduction of ABC or CARS score.

### 2.3. Study Selection and Data Extraction

#### 2.3.1. Selection of Literature Articles

After the exclusion of duplicate studies, two authors (Miran Bang and Sun-Ae Yu) independently reviewed titles and abstracts for the first exclusion. The full texts of the selected literature articles that potentially met the eligibility criteria were subjected to another review prior to the final selection of literature articles. Differences were resolved via discussion with the corresponding authors of this review (Gyu Tae Chang and Sang Yeon Min) in order to reach consensus.

#### 2.3.2. Data Extraction

One author (Miran Bang) conducted data extraction and another author (Sun Haeng Lee) reviewed the data. Items extracted from each study included author, publication year, sample size, patient age, diagnostic criteria, period of treatment, experimental and control intervention, outcomes, and ingredients of the herbal medicine.

### 2.4. Assessment of Risk of Bias

Two independent reviewers (Miran Bang and Kibong Kim) assessed methodological quality using the risk of bias (RoB) tool developed by Cochrane. Each study was assessed for selection bias (random sequence generation and allocation concealment), performance bias (blinding of participants and personnel), detection bias (blinding of outcome assessment), attrition bias (incomplete outcome data reporting), and reporting bias (selective outcome reporting). Each item of every included RCT was rated as “high risk,” “unclear,” or “low risk”; disagreements were resolved via discussion with other reviewers.

### 2.5. Data Analysis

Statistical analysis was performed using RevMan 5.3 analysis software (Cochrane Collaboration Review Manager Software). The impact of herbal medicines or herbal medicines in combination with other TCM treatment on dichotomous outcomes was expressed as a risk ratio (RR) with 95% confidence interval (CI). For continuous outcomes, mean difference (MD) with 95% CI was used.

## 3. Results

### 3.1. Study Selection and Description

**Figure 1:**
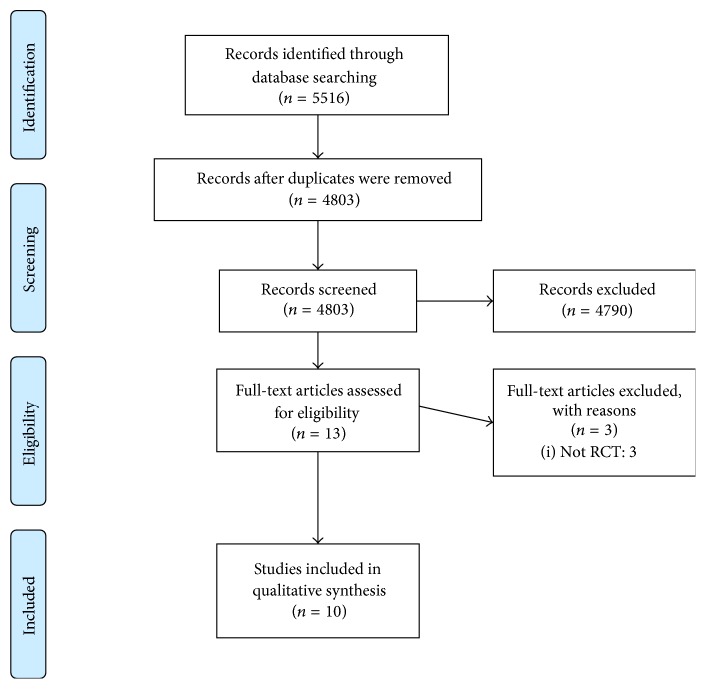
The PRISMA flow diagram of study selection.

A total of 5516 studies were initially retrieved: 588 studies in MEDLINE, 36 studies in AMED, 448 studies in EMBASE, 1559 studies in PsycARTICLES, 126 studies in the Cochrane Library, 196 studies in CINAHL, 899 studies in CNKI, 1455 studies in WANGFANG, 200 studies in CiNii, two studies in JAIRO, no studies in KoreaMed, 6 studies in OASIS, and 1 study in KTCKP. After removing 713 identical articles, 4803 studies were screened for eligibility. Among these, 4790 studies were excluded based on the title and abstract. Most of the studies were not related to herbal medicines intervention and were in vivo, in vitro, case reports, and retrospective studies; therefore, we could determine if the studies met inclusion criteria by inspecting only the title and abstract. After reviewing the full text of each article, 10 studies [14–23] involving 567 participants were included in this systematic review. The entire process was displayed by generating a flow diagram in Preferred Reporting Items for Systematic reviews and Meta-Analyses (PRISMA) (Figure 1).

The characteristics of the 10 studies are summarized in Table 1. The results of the included studies are summarized in Table 2. In eight studies [15–19, 21–23], participants were diagnosed using DSM-IV or the International Classification of Diseases version 10 (ICD-10). One study [14] did not report specific diagnostic criteria, and another study [20] used the ABC behavior scale, Klinefelter behavior scale, CARS scale, and clinical manifestations to diagnose ASD. All studies recruited only children. The treatment periods of the included studies were 1–6 months. Four studies [14, 16, 20, 23] evaluated herbal medicines as an adjuvant to conventional therapies, such as behavioral therapy, rehabilitation, and education, whereas one study [15] assessed herbal medicines combined with risperidone, a conventional medication. Various types of integrative therapy combined with conventional therapy were used in five studies [17–19, 21, 22]. In two studies [17, 21], herbal medicines plus acupuncture were used, Qiao et al. [18] assessed herbal medicines plus acupuncture and acupoint injection, Sun et al. [19] investigated herbal medicines plus acupuncture, acupoint injection, auricular acupoint massage, and acupoint catgut-embedding, and Zhao et al. [22] investigated herbal medicines plus acupuncture and Chuna therapy. The ingredients of herbal medicines used in the included RCTs are summarized in Table 3. The CARS score was reported in three studies [16, 19, 23], the ABC score was reported in one study [19], and the ABC-C score was reported in one study [15]. TER was reported in nine studies [14, 16–23].

### 3.2. Assessment of Risk of Bias

**Figure 2:**
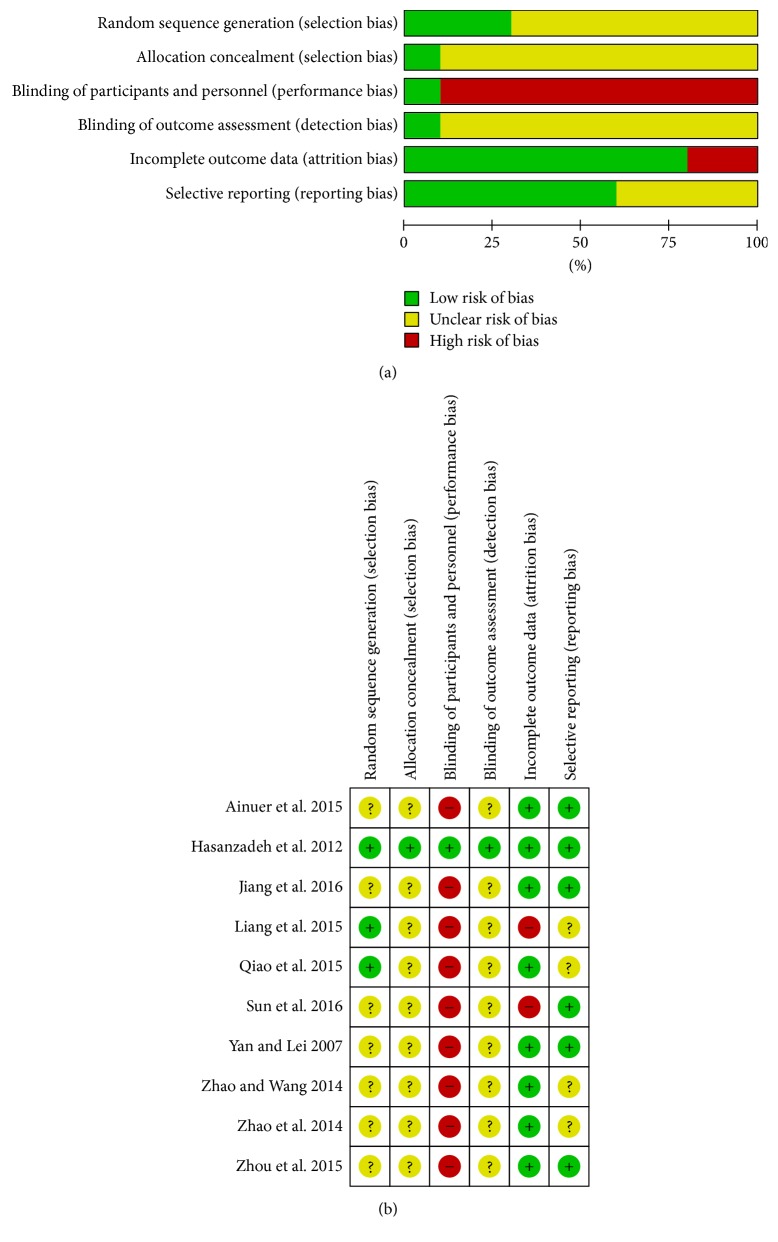
(a) Risk of bias graph: review of authors' judgements about each risk of bias item presented as percentages across all included studies. (b) Risk of bias summary: review of authors' judgements about each risk of bias item for each included study. “+”: low risk, “?”: unclear risk, and “−”: high risk.

Among 10 studies, three studies [15, 17, 18] reported the method of randomization and were rated with a low risk of bias, but the remaining studies [14, 16, 19–23] did not include the method of random sequence generation and were rated as unclear. One study [15], which used sealed, opaque envelopes, had a low risk of bias for allocation concealment, but the remaining studies were rated as unclear. Nine studies [14, 16–23] showed a high risk for blinding of participants and personnel and were also rated as unclear for blinding of outcome assessment. One study [15] showed a low risk of bias for blinding of participants, personnel, and outcome assessment. Two studies [17, 19] showed a high risk of bias for incomplete outcome data, because the studies did not include details of how the problem of dropout was resolved in statistical analysis. The remaining eight studies [14–16, 18, 20–23] showed a low risk of bias for incomplete outcome data. Four studies [17, 18, 21, 22] were rated as an unclear risk for selective reporting because the change in the CARS score was used in the criteria of TER, but the mean CARS score was not provided in the studies. Although we contacted a total of four corresponding authors of these studies via e-mail to obtain raw data, we received no replies. The remaining six studies [14–16, 19, 20, 23] that reported their outcomes using a previously described method or protocol had a low risk for selective reporting. The details of the risk of bias are provided in Figures 2(a) and 2(b).

### 3.3. Outcomes of the Included Studies

#### 3.3.1. CARS Score

Three RCTs [16, 19, 23] provided CARS scores. Of these three studies, two RCTs [16, 23] examined whether herbal medicines improved the CARS score when combined with conventional therapy. In the study of Jiang et al. [16], the administration of herbal medicines for 3 months showed significant effects on the CARS score when combined with conventional therapy (*n* = 60 participants, MD = −3.60, 95% CI: −7.00 to −0.20, *P* = 0.04). In the study of Zhou et al. [23], administration of herbal medicines for 3 months showed significant effects on CARS score when combined with conventional therapy (*n* = 60 participants, MD = −2.76, 95% CI: −5.20 to −0.32, *P* = 0.03) and for 6 months showed significant effects on CARS score (*n* = 60 participants, MD = −5.90, 95% CI: −8.50 to −3.30, *P* < 0.00001). The remaining study [19] examined whether the administration of herbal medicines for 3 months plus integrative therapy, including acupuncture, acupoint injection, auricular acupoint massage, and acupoint catgut-embedding, improved the CARS score when combined with conventional therapy. When herbal medicines plus integrative therapy were combined with conventional therapy, significant improvements were reported in the CARS score (*n* = 59 participants, MD = −3.59, 95% CI: −6.04 to −1.14, *P* = 0.004).

#### 3.3.2. ABC Score

Among the 10 studies, only one study [19] reported the ABC score. This study examined whether the administration of herbal medicines for 3 months plus integrative therapy, including acupuncture, acupoint injection, auricular acupoint massage, and acupoint catgut-embedding, improved the ABC score when combined with conventional therapy. When herbal medicines plus integrative therapy were combined with conventional therapy, significant improvements were reported in the ABC score (*n* = 59 participants, MD = −7.57, 95% CI: −12.12 to −3.02, *P* = 0.001).

#### 3.3.3. ABC-C Score

Among the 10 studies, one study [15] reported the ABC-C score. This study used five subscales of the ABC-C score to examine whether herbal medicines used as an adjuvant to conventional medication conferred additional benefits. In the present study, the experimental group was given* Ginkgo biloba* and risperidone for 10 weeks, while the control group received placebo and risperidone. The differences between the two groups were not significant, as indicated by the effect of groups-by-time interaction in all of the five subscales of the ABC-C score (Irritability Subscale: *F* = 1.72, df = 2.16, *P* = 0.18; Lethargy/Social Withdrawal Subscale: *F* = 0.24, df = 1.67, *P* = 0.74; Stereotypic Behavior Subscale: *F* = 0.95, df = 2.42, *P* = 0.40; Hyperactivity/Noncompliance Subscale: *F* = 0.26, df = 1.74, *P* = 0.73; Inappropriate Speech Subscale: *F* = 0.94, df = 1.84, *P* = 0.38).

#### 3.3.4. TER

Nine RCTs [14, 16–23] provided TER. Of these studies, four [14, 16, 20, 23] examined whether herbal medicines showed a significant increase in TER when combined with conventional therapy. In the study of Ainuer et al. [14], the administration of herbal medicines for 1 month showed no significant difference in TER when combined with conventional therapy (*n* = 21 participants, RR 1.24, 95% CI: 0.88 to 1.75, *P* = 0.22). In the study of Jiang et al. [16], the administration of herbal medicines for 3 months showed a significant increase in TER when combined with conventional therapy (*n* = 60 participants, RR 1.37, 95% CI: 1.01 to 1.86, *P* = 0.04). In the study of Yan and Lei [20], the administration of herbal medicines for 1 month showed a significant increase in TER when combined with conventional therapy (*n* = 37 participants, RR = 2.02, 95% CI: 1.01 to 4.02, *P* < 0.05). In the study of Zhou et al. [23], the administration of herbal medicines for 3 months showed a significant increase in TER when combined with conventional therapy (*n* = 60 participants, RR = 1.47, 95% CI: 1.03 to 2.09, *P* = 0.03), but the administration of herbal medicines for 6 months showed no significant difference in TER (*n* = 60 participants, RR = 1.07, 95% CI: 0.94 to 1.23, *P* = 0.31). The remaining five studies [17–19, 21, 22] examined whether administration of herbal medicines for 3 months plus integrative therapy improved TER when combined with conventional therapy. Of the five studies [17–19, 21, 22], two studies [17, 21] used herbal medicines plus acupuncture combined with conventional therapy in experimental group. In the study of Liang et al. [17], a significant increase in TER was reported (*n* = 67 participants, RR = 2.06, 95% CI: 1.30 to 3.27, *P* = 0.002). In the study of Zhao and Wang [21], a significant increase in TER was also reported (*n* = 60 participants, RR = 1.53, 95% CI: 1.09 to 2.16, *P* = 0.02). When herbal medicines plus integrative therapy, including acupuncture and acupoint injection, were combined with conventional therapy, significant differences were observed in TER (*n* = 84 participants, RR = 1.38, 95% CI: 1.11 to 1.71, *P* = 0.003) [18]. When herbal medicines plus integrative therapy, including acupuncture and Chuna therapy, were combined with conventional therapy, a significant increase was reported in TER (*n* = 72 participants, RR = 1.41, 95% CI: 1.05 to 1.89, *P* = 0.02) [22]. When herbal medicines plus integrative therapy, including acupuncture, acupoint injection, auricular acupoint massage, and acupoint catgut-embedding, were combined with conventional therapy, no significant differences were observed in TER (*n* = 59 participants, RR = 1.29, 95% CI: 0.97 to 1.73, *P* = 0.08) [19].

### 3.4. Adverse Events

Among the 10 RCTs, eight studies [14, 16–18, 20–23] did not record information on the occurrence of adverse events. Of the remaining two studies, one study [19] reported that none of the participants had experienced adverse events, and another study [15] reported that there was no significant difference in the incidents of side effects such as daytime drowsiness, increased appetite, and nervousness between the experimental group receiving* G. biloba* plus risperidone and the control group receiving risperidone alone. These adverse events were thought to be associated with the administration of risperidone in both the experimental and control groups, because the authors of the study mentioned that* G. biloba* was relatively safe.

## 4. Discussion

### 4.1. Summary of Evidence

In the present study, we analyzed 10 RCTs involving 567 individuals to assess the efficacy of herbal medicines for the treatment of ASD. Because of the high risk of bias for blinding of participants observed in the included studies, diversity of herbal medicines, and an insufficient number of the studies included, meta-analysis was not performed in this review. Based on the findings in this systematic review, herbal medicines and herbal medicines plus integrative therapy can significantly improve the CARS score, which measures the core autistic features in children with ASD, when combined with conventional therapy. In one study, herbal medicines plus integrative therapy significantly improved ABC score when combined with conventional treatment. Herbal medicines had no beneficial effects on the ABC-C scale score when combined with risperidone in one study. When herbal medicines were combined with conventional therapy, two [16, 20] of the four studies [14, 16, 20, 23] showed a significant increase in TER and one study [14] showed no significant difference in TER. In the remaining study [23], the administration of herbal medicines for 3 months showed a significant increase in TER, but a 6-month administration showed no significant difference in TER. This was thought to be because there was significant difference between experimental and control group by 3 months, but after that time, the TER of the control group also increased; finally, no significant difference was observed between the two groups by 6 months. Herbal medicines plus integrative therapy in four of the five studies showed a significant increase in TER. Within the studies documenting the adverse events, no serious adverse events associated with herbal medicines were observed. Conclusions regarding the safety of herbal medicines and herbal medicines plus integrative therapy could not be drawn owing to the paucity of evidence reported by the included studies.

### 4.2. Pharmacological and Clinical Effects of Herbal Medicines Used in the Included Studies

Among the 10 studies, the commonly used herbal medicines included* Poria cocos, Panax ginseng, Acorus gramineus, Schisandra chinensis, *and* Glycyrrhiza uralensis. *One study reported that* P. ginseng *improved abnormal behaviors in animal models of autism [24].* A. gramineus*, which has various pharmacological effects such as sedative, antispasmodic, and anticonvulsant activities, is used for the treatment of various pediatric aliments such as cough, epilepsy, abdominal pain, and mental diseases, including psychoneurosis, schizophrenia, insomnia, and loss of memory [25].* S. chinensis* was reported to have sedative and hypnotic activities, which might be mediated via the control of the serotonergic system [26].* P. cocos* is a well-known herbal medicine used for its sedative and tonic effects [27]. These herbal medicines may contribute to the improvement of abnormal behaviors, inattention, or seizures in autistic children. However, further research should be conducted to demonstrate the specific pharmacological mechanisms of treating autism and to examine whether herbal medicines exhibit pharmacological activities as polyherbal formulations.

### 4.3. Comparison with Other Systematic Reviews

In 2015, a systematic review revealed effective Chinese herbal medicines and provided evidence for autism treatment by analyzing modern literature, ancient books, and monographs [8]. The study concluded that TCM used a holistic treatment strategy with comprehensive care and the pharmacological activities of 32 types of Chinese herbal medicines in the treatment of ASD. However, this study did not evaluate the clinical efficacy of herbal medicines in the treatment of children with ASD. In our systematic review, we managed to summarize all published RCTs to assess the clinical efficacy of herbal medicines for the treatment of ASD in children. The findings of our systematic review suggested that herbal medicines and herbal medicines plus integrative therapy improved the CARS score, and herbal medicines plus integrative therapy showed further significant effects on TER when combined with conventional treatment.

### 4.4. Limitations

The present systematic review has several limitations. First, most of the included studies had a relatively low methodological quality. Of the 10 RCTs, only 3 described a randomization method, 1 included the allocation method, and only 1 had a double-blind design. Thus, there might have been a possibility that the clinical effects of herbal medicines for the treatment of ASD have been overestimated. Second, in nine studies [14, 16–23], with the exclusion of one study [15], a placebo identical to herbal medicines was not used and conventional therapy was concurrently used in both the experimental and control groups. Therefore, the positive effects cannot be solely attributed to the efficacy of herbal medicines. Third, the tested herbal medicines varied in terms of the composition and duration of treatment. Because of this diversity of herbal medicines, a meta-analysis for the evaluation of the effects of herbal medicines could not be performed. Additionally, sensitivity analysis and tests for publication bias could not be conducted because there were an insufficient number of studies with a high methodological quality among the included trials. Finally, this review may have potential publication or location biases; of the 10 RCTs, 1 was conducted in Iran and the remaining 9 were performed in China.

### 4.5. Suggestions for Future Research

The RCTs included in the present systematic review comprised low methodological qualities and it was confirmed that the conclusions drawn from this review are somewhat limited owing to methodological deficiencies. Future trials should use rigorous randomization and blinding methods and provide details of the methods. In addition, future studies should report the incidence of adverse events associated with herbal medicines. Given the difficulty to diagnose ASD especially at younger age, future studies should use international criteria and adopt standardized assessment tools, such as Autism Diagnostic Interview-Revised (ADI-R) and Autism Diagnostic Observation Schedule (ADOS), for the diagnosis and assessment of autism [28, 29]. Considering the diversity of herbal medicines and varieties of integrative therapy combined with herbal medicines in this review, future research should standardize the optimal composition of herbal medicines and types of integrative therapy. This standardization will improve the applicability and generalization of herbal medicine treatment for children with ASD.

## 5. Conclusion

The results of this systematic review indicated that herbal medicines combined with conventional treatment seem to have a positive effect on the treatment of ASD in children. Herbal medicines plus integrative therapy as an adjuvant to conventional therapy also have an encouraging effect in the treatment of autistic children. However, owing to the low methodological quality of the included studies, small sample size, and diversity of herbal medicines, firm conclusions could not be drawn. Further well-designed, large-scale RCTs, which have a low risk of bias, are needed to confirm these results.

## Supplementary Material

Search Strategy Used in English Databases.

## Figures and Tables

**Table 1:** Characteristics of the included studies.

Author, year	Sample size (E/C)	Age (mean or range)	Diagnosis of ASD	Experimental intervention	Control intervention	Period	Study design	Outcomes
Ainuer et al., 2015 [14]	21 (11/10)	E: 3–8 years C: 3–8 years	Not reported of specific diagnostic criteria	(1) Jiawei Wendan decoction (b.i.d.) (2) Control intervention	ABA training and guided education	1 month	HMs + conventional therapy versus conventional therapy	(1) TER

Hasanzadeh et al., 2012 [15]	47 (23/24)	E: 6.04 ± 1.61 (4–10) years C: 6.76 ± 2.60 (4–11) years	DSM-IV	(1) *Ginkgo biloba* (40 mg b.i.d. for patients under 30 kg and 60 mg b.i.d. for patients above 30 kg) (2) Risperidone (1–3 mg/day)	(1) Placebo (2) Risperidone (1–3 mg/day)	10 weeks	HMs + conventional therapy versus conventional therapy	(1) ABC-C score

Jiang et al., 2016 [16]	60 (30/30)	E: 5.93 ± 2.28 years C: 6.10 ± 2.26 years	DSM-IV	(1) Modified Yinhuo decoction (150 cc b.i.d.) (2) Control intervention	Training	3 months (3 courses of 30 days each)	HMs + conventional therapy versus conventional therapy	(1) TER (2) CARS score

Liang et al., 2015 [17]	67 (33/34)	E: 3.36–4.28 years C: 3.37–4.29 years	DSM-IV, ICD-10	(1) Suhe Ditan decoction: Suhexiang wan (0.75 g b.i.d.) + Ditan decoction (b.i.d.) (2) Acupuncture (3) Control intervention	Rehabilitation training	3 months	HMs + integrative therapy + conventional therapy versus conventional therapy	(1) TER

Qiao et al., 2015 [18]	84 (42/42)	E: 3.7 ± 2.18 years C: 3.8 ± 1.74 years	ICD-10, TCM diagnostic criteria	(1) Jingshuaikang capsule, Congnaoyizhi capsule (2) Acupuncture (3) Acupoint injection (Compound Danshen Injection 2 mL, Compound Musk Injection 2 mL) (4) Control intervention	Education training	3 months	HMs + integrative therapy + conventional therapy versus conventional therapy	(1) TER

Sun et al., 2016 [19]	59 (29/30)	E: 2–6 (6.5) years C: 2–6 (6.5) years	ICD-10, TCM diagnostic criteria	(1) Jingshuaikang capsule 0.3 g (2-3 years: 0.9 g t.i.d., 3–7 years: 1.2 g t.i.d., over 7 years: 1.5–1.8 g t.i.d.) or with Congnaoyizhi capsule 0.3 g (2-3 years: 0.9 g t.i.d., over 3 years: 1.2 g t.i.d.) (2) Acupuncture (3) Acupoint injection (Compound Danshen Injection 2 mL, Compound Musk Injection 2 mL) (4) Auricular acupoint massage (5) Acupoint embedding (6) Control intervention	Rehabilitation	3 months	HMs + integrative therapy + conventional therapy versus conventional therapy	(1) TER (2) ABC score (3) CARS score (4) Gesell child growth scale

Yan and Lei, 2007 [20]	37 (25/12)	E: 3–8 years C: 3–8 years	Observed by ABC behavior scale, Klinefelter behavior scale, CARS scale, clinical manifestations of autistic children	(1) Jiawei Wendan decoction (b.i.d.) (2) Control intervention	ABA training and guided education	1 month	HMs + conventional therapy versus conventional therapy	(1) TER

Zhao and Wang, 2014 [21]	60 (30/30)	E: 1.8–6 years C: 2.6–6 years	ICD-10, TCM diagnostic criteria	(1) Jingshuaikang capsule 0.3 g (2.5–3 years: 0.6 g t.i.d., 3–6 years: 0.9 g t.i.d.) and Congnaoyizhi capsule 0.3 g (2.5–3 years: 0.6 g t.i.d., 3–6 years: 0.9 g t.i.d.) (2) Acupuncture (3) Control intervention	Rehabilitation training	3 months	HMs + integrative therapy + conventional therapy versus conventional therapy	(1) TER

Zhao et al., 2014 [22]	72 (36/36)	E: 2.7–6.6 years C: 2.3–6 years	DSM-IV and ICD-10	(1) Canrongjiannao capsule 0.3 g (2.5–3 years: 0.6 g t.i.d., 3–6 year: 0.9 g t.i.d.) (2) Acupuncture (3) Chuna treatment (4) Control intervention	Language and behavior training	3 months	HMs + integrative therapy + conventional therapy versus conventional therapy	(1) TER

Zhou et al., 2015 [23]	60 (30/30)	E: 4.75 ± 1.03 years C: 4.98 ± 1.99 years	DSM-IV	(1) Supplemented Lizhong decoction (150 cc t.i.d.) (2) Control intervention	Education, behavioral therapy	6 months (1 course: 21-day treatment period + 9-day rest period; total 6 courses)	HMs + conventional therapy versus conventional therapy	(1) TER (2) CARS score

*Note.* E: experimental group; C: control group; b.i.d.: twice a day; ABA: Applied Behavior Analysis; HMs: herbal medicines; TER: total effective rate; ABC-C: Aberrant Behavior Checklist-Community; CARS: Childhood Autism Rating Scale; ICD-10: International Classification of Diseases version 10; TCM: Traditional Chinese Medicine; t.i.d.: three times a day; ABC: Autism Behavior Checklist.

**Table 2:** Results of the included studies.

Author, year	Effect size^*∗*^
Ainuer et al., 2015 [14]	(1) TER: 1.24 [0.88, 1.75], *P* = 0.22

Hasanzadeh et al., 2012 [15]	(1) ABC-C score: (i) Irritability: 0.66 [−3.10, 4.42], *P* = 0.73 (ii) Lethargy and social withdrawal: −0.50 [−3.99, 2.99], *P* = 0.78 (iii) Stereotypic behavior: −0.30 [−9.93, 9.33], *P* = 0.95 (iv) Hyperactivity and noncompliance: 1.70 [−2.58, 5.98], *P* = 0.44 (v) Inappropriate speech: −0.35 [−1.27, 0.57], *P* = 0.46

Jiang et al., 2016 [16]	(1) TER: 1.37 [1.01, 1.86], *P* = 0.04 (2) CARS score: −3.60 [−7.00, −0.20], *P* = 0.04

Liang et al., 2015 [17]	(1) TER: 2.06 [1.30, 3.27], *P* = 0.002

Qiao et al., 2015 [18]	(1) TER: 1.38 [1.11, 1.71], *P* = 0.003

Sun et al., 2016 [19]	(1) TER: 1.29 [0.97, 1.73], *P* = 0.08 (2) ABC score: −7.57 [−12.12, −3.02], *P* = 0.001 (3) CARS score: −3.59 [−6.04, −1.14], *P* = 0.004

Yan and Lei, 2007 [20]	(1) TER: 2.02 [1.01, 4.02], *P* < 0.05

Zhao and Wang, 2014 [21]	(1) TER: 1.53 [1.09, 2.16], *P* = 0.02

Zhao et al., 2014 [22]	(1) TER: 1.41 [1.05, 1.89], *P* = 0.02

Zhou et al., 2015 [23]	(1) TER: (i) 3 months: 1.47 [1.03, 2.09], *P* = 0.03 (ii) 6 months: 1.07 [0.94, 1.23], *P* = 0.31 (2) CARS score: (i) 3 months: −2.76 [−5.20, −0.32], *P* = 0.03 (ii) 6 months: −5.90 [−8.50, −3.30], *P* < 0.00001

*Note.∗* is showed as TER: RR [95% CI], *P* value; CARS, ABC-C, or ABC score: MD [95% CI], *P* value; TER: total effective rate; ABC-C: Aberrant Behavior Checklist-Community; RR: risk ratio; MD: mean difference; 95% CI: 95% confidence interval; CARS: Childhood Autism Rating Scale; ABC: Autism Behavior Checklist.

**Table 3:** Composition of herbal medicines in the included RCTs.

Author, year	Intervention	Composition	Formulation
Ainuer et al., 2015 [14]	Jiawei Wendan decoction	*Glycyrrhiza uralensis *3 g, *Bambusa tuldoides *2 g, *Citrus aurantium* 5 g, *Pinellia ternate* 7 g, *Citrus reticulate* 6 g, *Codonopsis pilosula* 7 g, *Alpinia oxyphylla* 6 g, *Zingiber officinale* 3 g, *Acorus gramineus* 6 g	Decoction

Hasanzadeh et al., 2012 [15]	*Ginkgo biloba*	*Ginkgo biloba*. Amount was not specified	Pill

Jiang et al., 2016 [16]	Modified Yinhuo decoction	*Rehmannia glutinosa *60–120 g, *Morinda officinalis* 15–30 g, *Asparagus cochinchinensis* 15–30 g, *Ophiopogon japonicas* 15–30 g, *Poria cocos* 10–30 g, *Schisandra chinensis* 5–10 g, *Cinnamomi cortex *3–6 g	Decoction

Liang et al., 2015 [17]	Suhe Ditan decoction	Suhexiang wan + Ditan decoction Suhexiang wan: *Liquidambar orientalis*, *Moschus berezovskii*, *Blumea balsamifera*, *Styrax tonkinensis*, *Aucklandia lappa*, *Santalum album*, *Aquilaria sinensis*, *Boswellia carteri*, *Syzygium aromaticum*, *Cyperus rotundus*, *Piper longum*, *Atractylodes macrocephala*, *Terminalia chebula*, *Bubalus bubalis*, *Cinnabaris*. Amounts were not specified Ditan decoction:* Poria cocos* 6 g, *Panax ginseng* 3 g, *Citrus reticulate* 6 g,* Bile arisaema* 3 g, *Pinellia ternate* 8 g, *Bambusa tuldoides *2 g, *Citrus aurantium* 6 g, *Acorus calamus* 3 g, *Zingiber officinale* 3 g, *Ziziphus jujuba* 3 g,* Glycyrrhiza uralensis* 2 g	Pill and decoction

Qiao et al., 2015 [18]	Jingshuaikang capsule, Congnaoyizhi capsule	(1) Jingshuaikang capsule: *Gastrodia elata*, *Paeonia lactiflora*, *Bubalus bubalis*, *Ziziphus jujuba*, *Schisandra chinensis*, *Curcuma longa*, *Glycyrrhiza uralensis*. Amount was not specified (2) Congnaoyizhi capsule: *Polygala tenuifolia*,* Acorus gramineus, Panax ginseng*, *Poria cocos, Cinnamomi cortex, Cervus nippon*,* Cinnamomi ramulus, Angelica sinensis, Zingiber officinale*, *Paeonia lactiflora*, *Ligusticum striatum*, *Glycyrrhiza uralensis*. Amounts were not specified	Capsule

Sun et al., 2016 [19]	Jingshuaikang capsule or with Congnaoyizhi capsule	(1) Jingshuaikang capsule: *Gastrodia elata*, *Paeonia lactiflora*, *Bubalus bubalis*, *Ziziphus jujuba*, *Schisandra chinensis*, *Curcuma longa*, *Glycyrrhiza uralensis*. Amounts were not specified (2) Congnaoyizhi capsule: *Polygala tenuifolia*,* Acorus gramineus, Panax ginseng*, *Poria cocos, Cinnamomi cortex, Cervus nippon*,* Cinnamomi ramulus, Angelica sinensis, Zingiber officinale*, *Paeonia lactiflora*, *Ligusticum striatum*, *Glycyrrhiza uralensis*. Amounts were not specified	Capsule

Yan and Lei, 2007 [20]	Jiawei Wendan decoction	*Citrus reticulate* 5 g, *Pinellia ternate* 6 g, *Poria cocos* 6 g, *Glycyrrhiza uralensis* 2 g, *Bambusa tuldoides *1 g, *Citrus aurantium* 4 g, *Codonopsis pilosula* 6 g, *Acorus gramineus *5 g,* Alpinia oxyphylla* 5 g, *Zingiber officinale *2 g	Decoction

Zhao and Wang, 2014 [21]	Jingshuaikang capsule, Congnaoyizhi capsule	(1) Jingshuaikang capsule: *Gastrodia elata*, *Paeonia lactiflora*, *Bubalus bubalis*, *Ziziphus jujuba*, *Schisandra chinensis*, *Curcuma longa*, *Glycyrrhiza uralensis*. Amounts were not specified (2) Congnaoyizhi capsule: *Polygala tenuifolia*,* Acorus gramineus*, *Panax ginseng*, *Poria cocos, Cinnamomi cortex, Cervus nippon*,* Cinnamomi ramulus, Angelica sinensis, Zingiber officinale*, *Paeonia lactiflora*, *Ligusticum striatum*, *Glycyrrhiza uralensis*. Amounts were not specified	Capsule

Zhao et al., 2014 [22]	Canrongjiannao capsule	*Astragalus membranaceus*,* Panax ginseng*,* Poria cocos*, *Cervi Parvum Cornu*, *Zingiber officinale*,* Angelica sinensis*, *Eucommia ulmoides*, *Cinnamomi ramulus*, *Paeonia lactiflora, Pinellia ternate*, *Cuscuta chinensis*, *Glycyrrhiza uralensis*. Amounts were not specified	Capsule

Zhou et al., 2015 [23]	Supplemented Lizhong decoction	*Zingiber officinale *15 g,* Panax ginseng *15 g, *Glycyrrhiza uralensis *15 g, *Atractylodes macrocephala *30 g, *Prunus mume *9 g,* Schisandra chinensis *5 g	Decoction
