# Supplementary material for: Herbal Medicine Treatment for Children with Autism Spectrum Disorder: A Systematic Review
Source: Evid Based Complement Alternat Med. 2017 May 16;2017:8614680. doi: 10.1155/2017/8614680 (PMC5448044; doi:10.1155/2017/8614680)
Supplement: Supplementary file 1 — Search Strategy Used in English Databases. [file 8614680.f1.pdf]

Supplementary Material (Supplement 1)

Search Strategy Used in English Databases

MEDLINE, Cochrane Library, Cumulative Index to Nursing and Allied Health Literature (CINAHL), AMED, PsycArticles

(autis* OR pervasive developmental disorder* OR childhood disintegrative disorder OR Asperger* OR Autism Spectrum Disorder OR Child Development Disorders, Pervasive) AND (herb* OR decoction* OR remed* OR Chinese medic* OR Korean medi* OR kampo OR formul* OR herbal drug* OR Chinese drug* OR plant* OR Chinese prescrip* OR Chinese materica* medica* OR traditional medic* OR Medicine, East Asian Traditional OR Herbal Medicine)

EMBASE

('autism'/exp OR 'autism' OR 'asperger syndrome'/exp OR 'asperger syndrome' OR 'autism spectrum disorder' OR 'child development disorders, pervasive') AND (‘herb’/exp OR 'herb' OR ‘herbal medicine’/exp OR ‘herbal medicine’ OR ‘Chinese herb’/exp OR ‘Chinese herb’ OR ‘traditional medicine’/exp OR ‘traditional medicine’ OR ‘Chinese medicine’/exp OR ‘Chinese medicine’ OR ‘Korean medicine’/exp OR ‘Korean medicine’ OR ‘kampo’/exp OR ‘kampo’ OR ‘herbaceous agent’/exp OR ‘herbaceous agent’ OR ‘Chinese drug’/exp OR ʻChinese drug’ OR ‘plant’/exp OR ‘plant’ OR ‘Medicine, East Asian Traditional’ OR ‘Herbal Medicine’)
